# Early Movement Restriction Affects FNDC5/Irisin and BDNF Levels in Rat Muscle and Brain

**DOI:** 10.3390/ijms25073918

**Published:** 2024-03-31

**Authors:** Orlane Dupuis, Julien Girardie, Mélanie Van Gaever, Philippe Garnier, Jacques-Olivier Coq, Marie-Hélène Canu, Erwan Dupont

**Affiliations:** 1 Univ. Lille, Univ. Artois, Univ. Littoral Côte d’Opale, ULR 7369, URePSSS—Unité de Recherche Pluridisciplinaire Sport Santé Société, F-59000 Lille, Francejulien.girardie@univ-lille.fr (J.G.); erwan.dupont@univ-lille.fr (E.D.); 2 INSERM UMR1093-CAPS, Université Bourgogne Franche-Comté, UFR Des Sciences de Santé, F-21000 Dijon, France; 3 Département Génie Biologique, IUT, F-21000 Dijon, France; 4 Institut des Sciences du Mouvement, UMR7287, Aix-Marseille Université, F-13000 Marseille, France; jacques-olivier.coq@univ-amu.fr; 5 Centre National de la Recherche Scientifique (CNRS), UMR7287, F-13000 Marseille, France

**Keywords:** muscle–brain dialogue, cerebrospinal fluid, blood, myokines, development

## Abstract

Interaction with the environment appears necessary for the maturation of sensorimotor and cognitive functions in early life. In rats, a model of sensorimotor restriction (SMR) from postnatal day 1 (P1) to P28 has shown that low and atypical sensorimotor activities induced the perturbation of motor behavior due to muscle weakness and the functional disorganization of the primary somatosensory and motor cortices. In the present study, our objective was to understand how SMR affects the muscle–brain dialogue. We focused on irisin, a myokine secreted by skeletal muscles in response to exercise. FNDC5/irisin expression was determined in hindlimb muscles and brain structures by Western blotting, and irisin expression in blood and cerebrospinal fluid was determined using an ELISA assay at P8, P15, P21 and P28. Since irisin is known to regulate its expression, Brain-Derived Neurotrophic Factor (BDNF) levels were also measured in the same brain structures. We demonstrated that SMR increases FNDC5/irisin levels specifically in the soleus muscle (from P21) and also affects this protein expression in several brain structures (as early as P15). The BDNF level was increased in the hippocampus at P8. To conclude, SMR affects FNDC5/irisin levels in a postural muscle and in several brain regions and has limited effects on BDNF expression in the brain.

## 1. Introduction

The first 1000 days of life is a concept introduced by Unicef in 2017, which argues that the period from conception to the first two years of a child’s life is a sensitive one. In this period, various factors, both positive and negative, influence short- and long-term health. Indeed, early childhood is a period of body construction and brain development, with the highest level of synaptogenesis of all life, during which sensorimotor and cognitive functions are acquired. The maturation and refinement of the central nervous system is highly sensitive to physical and social interactions. In particular, sensorimotor experience can shape neuronal circuits and ultimately drive the maturation of brain functions (for review: [1]).

A reduction in physical activity and low-level interactions with the environment may be the consequence of accidents or illnesses requiring prolonged bed rest. Hypoactivity is also a common situation in children with developmental coordination disorder [2,3]. This neurodevelopmental disorder, which concerns approximately 5–6% of school-aged children [4], mainly corresponds to motor impairments ranging from gross- to fine-skill deficits that interfere with daily social and academic activities [5]. Developmental coordination disorder results in an atypical sensorimotor experience and poor interaction with the environment affecting the entire sensorimotor pathway. More commonly, sedentary behavior increases in children, even in the youngest of them. Studies conducted in infants are rare, but one recent paper has shown that few infants met the individual recommendations for physical activity and sedentary behavior (screen time and reading time) in the first 6 months of life [6].

To study the effects of early life hypoactivity, a postnatal sensorimotor restriction (SMR) model was developed in rats. In this model, animals are subject to an atypical sensorimotor activity from postnatal day 1 (P1) to P28 by hindlimb immobilization, over 16 h/day. This sensorimotor restriction induces persistent muscle atrophy characterized by a reduction in the fiber area, a loss of strength, persistence of neonatal and fast-myosin heavy-chain isoforms at the expense of the slow ones, drastic alterations in motor coordination, hyperreflexia of the lumbar spinal cord, cortical disorganization with a reduction of the cortical representation maps of the hindlimb in both the primary sensory and motor cortices, alterations of cortical neuron properties and spinal and cortical hyperexcitability [7,8,9].

Due to the reciprocal interplay between the muscle and the central nervous system, sensorimotor restriction generates an atypical sensory input that affects the somatosensory pathway. In turn, the motor command is altered, which affects muscle properties [10]. Thus, brain and muscles communicate through a deleterious, self-sustaining cycle: abnormal movements affect the immature nervous system. This situation affects the motor command, which in turn has consequences on the muscular system, reinforcing the production of abnormal movements [10]. In addition to this sensorimotor neuronal pathway, the muscles and brain also communicate through the endocrine way. Indeed, human and animal studies have shown that skeletal muscle is a secretory organ that produces, expresses and releases molecules classified as “myokines” [11], which exert autocrine, paracrine or endocrine effects. Among the myokines, several studies have shown that irisin is secreted by skeletal muscles in response to physical activity [11,12,13,14]. This polypeptide is secreted in the plasma after the cleavage of the Fibronectin Type III Domain Containing 5 protein (FNDC5), a membrane protein present in muscle. Fndc5 gene expression is regulated by the peroxisome proliferator-activated receptor coactivator-1α (PGC-1α) [12], which is known to mediate the effects of exercise on muscle.

Known and widely studied for its role in the browning of white adipocytes [12], irisin exerts pleiotropic effects on many organs including the liver, pancreas, bone tissue and cardiovascular system (for review: [15]). Irisin can also cross the blood–brain barrier and is, therefore, thought to mediate the beneficial effects of exercise in the brain [16,17]. It also appears to protect against cognitive impairment induced by neurodegenerative pathologies [16,17], exerts antidepressant effects [18] and limits ischemia-induced brain damage [19]. In the hippocampus, irisin induces the expression of the Brain-Derived Neurotrophic Factor (BDNF) [13,20], a pivotal neurotrophin for synaptic plasticity and cognitive functions. Taken together, these data suggest that irisin is an important messenger in the muscle–brain dialogue. The effects of physical activity on myokine levels are well documented in adults; yet, there are very little data on the effects of hypoactivity, particularly during development. Our hypothesis is that, in contrast to the effects of exercise, the irisin level will be lower in SMR pups.

In the present paper, our objective was to determine whether early SMR in rats affects the muscle–brain dialogue by investigating FNDC5/Irisin expression in hindlimb muscles (the slow postural soleus (SOL) muscle and the fast extensor digitorum longus (EDL) and tibialis anterior (TA)) and various brain structures involved in motor (sensorimotor cortex and striatum) or cognitive functions (prefrontal cortex and hippocampus), as well as irisin expression in blood and cerebrospinal fluid (CSF). BDNF levels were also measured in the same brain structures. The measurements were realized at several developmental stages (P8, P15, P21 and P28) and in both sexes to investigate both possible age- and sex-dependent effects. Our results show that SMR increases FNDC5/irisin levels in the postural soleus muscle, but has only limited effects on BDNF expression in the brain.

## 2. Results

Initially, we analyzed FNCD5/irisin and BDNF expression in skeletal muscle and brain structures from both male and female individuals. Most data showed no difference in expression between sexes. In consequence, males and females were pooled. However, the ANOVA analysis of the sex and group effects is available in Supplementary Tables S1–S4 and the sex effects are specified in the results for each analysis.

### 2.1. Impact of SMR on Hindlimb Muscle Weight

**Figure 1:**
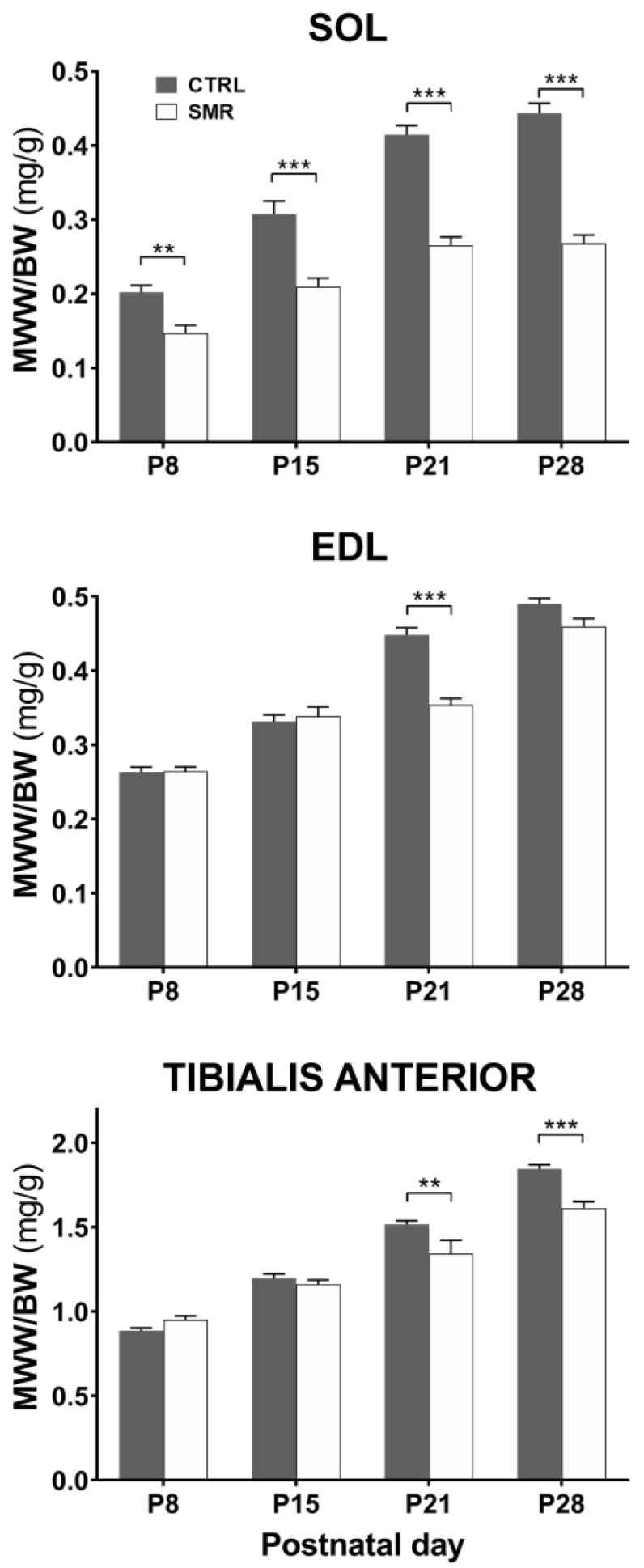
Effects of SMR on hindlimb muscle weight at different ages. MWW/BW ratio for SOL, EDL and Tibialis anterior muscles at P8, P15, P21 and P28. Muscles were sampled in 20 CTRL and 20 SMR at each age. Values are the mean ± SEM. **: *p* < 0.01, ***: *p* < 0.001 with respect to CTRL. The SOL data were reproduced with permission from Dupuis, Brain research; published by Elsevier, 2024.

In a series of rats, we assessed whether SOL, EDL and TA muscles were atrophied as soon as P8 and until P28 (Figure 1). We observed a drastic decrease in SOL muscle wet weight (MWW) relative to body weight (BW) in SMR rats [Group effect: F(1,167) = 186, *p* < 0.0001]. SMR rats displayed a severe atrophy of SOL muscle at P8 (−27%, t = 3.649, *p* < 0.01) and atrophy increased over time (P15: −32%, t = 5.421, *p* < 0.001; P21: −36%, t = 8.148, *p* < 0.001; P28: −39%, t = 9.559, *p* < 0.001). A group effect was also detected in EDL [F(1,168) = 22, *p* < 0.0001] and TA muscles [F(1,168) = 15, *p* < 0.001]. However, the decrease in the MWW/BW ratio was less pronounced than in SOL muscle and only at P21 for EDL (−21%, t = 7.244, *p* < 0.0001) and P21 and P28 for TA (−11% t = 3.478 *p* = 0.0026 and −13%, t = 4.632 *p* < 0.0001 respectively). In addition, no sex effect was detected (Supplementary Table S1).

### 2.2. Differential Impact of SMR on FNDC5/Irisin Levels in Hindlimb Muscles

**Figure 2:**
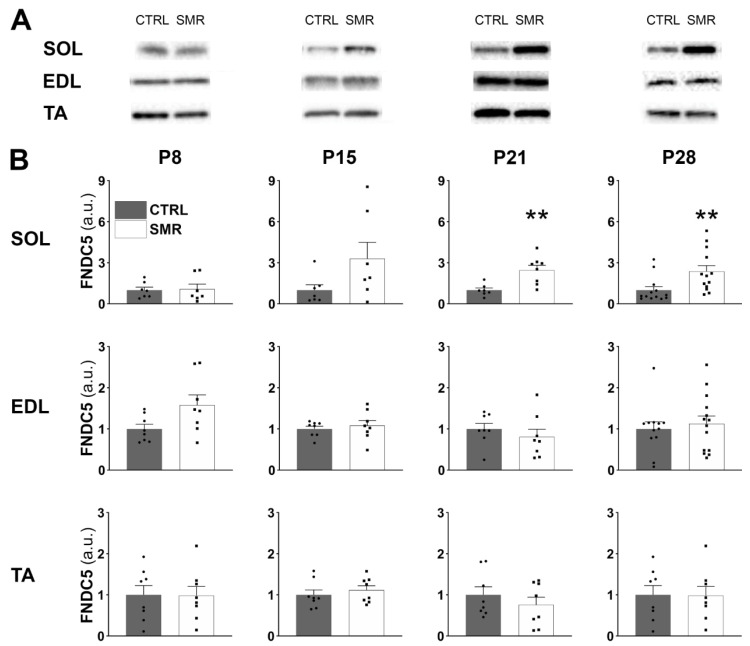
Effects of SMR on FNDC5/irisin levels in hindlimb muscles at different ages. Muscle FNDC5/irisin level was determined by Western blotting (**A**). FNDC5/irisin expression was calculated as the ratio FNDC5/whole proteome (stain free) (**B**). a.u. = arbitrary unit. Each point represents the value for a given animal. Data are expressed as mean ± S.E.M. **, *p* < 0.01 vs. CTRL (Mann–Whitney).

In SOL (Figure 2), the FNDC5/irisin level was similar between CTRL and SMR animals at P8 (*p* = 0.9999). At P15, the level in SMR pups was three-fold that of CTRL pups, although the difference was not significant due to the high variability between subjects (*p* = 0.1649). At P21 and P28, the FNDC5/irisin level was significantly increased in SMR animals (x2.5 approximately), compared to CTRL animals (*p* = 0.0037 and 0.0019, respectively). In EDL and TA, no difference between the groups was noticed whatever the age.

**Figure 3:**
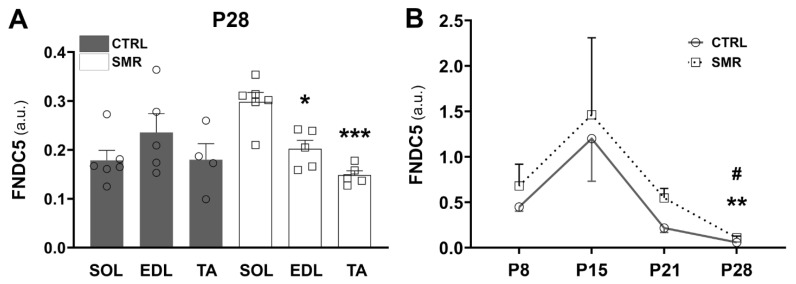
FNCD5/irisin level in various muscles at P28 (**A**) and in soleus muscle at several developmental stages (**B**). Muscle FNDC5/irisin expression was determined by Western blotting and was calculated as the ratio FNDC5/whole proteome (stain free). a.u. = arbitrary unit. Each point represents the value for a given animal. Data are expressed as mean ± S.E.M. (**A**) Values were compared by a two-way ANOVA followed by Tukey’s test. * *p* < 0.05, *** *p* < 0.01 vs. SOL SMR. (**B**) Values were compared by a Kruskal–Wallis followed by Dunn’s test. ** *p* < 0.01 (P15 CTRL vs. P28 CTRL), # *p* < 0.05 (P15 SMR vs. P28 SMR).

We also compared the FNDC5/irisin levels between hindlimb muscles (SOL, EDL and TA) at P28 to determine whether protein levels differ with the muscle type (Figure 3A). Two-way ANOVA revealed a muscle effect [F(2.25) = 4.81, *p* < 0.05] and a muscle × group interaction [F(2.25) = 7.09, *p* < 0.01]. For CTRL animals, there was no difference between the hindlimb muscles. However, the FNDC5/irisin value differed between muscles for SMR animals, with a higher expression of FNCD5 in SOL compared to TA (+99%, *p* = 0.0003) and EDL (+47%, *p* = 0.0198).

No sex effect was detected in the SOL muscle. In contrast, in the EDL muscle, FNDC5/irisin expression was higher in females at P8 (*p* < 0.05) and P21 (*p* < 0.01), whereas it was lower in the TA muscle at P15 (*p* < 0.05) (Supplementary Table S2).

We then analyzed the expression kinetics of FNDC5/irisin expression from P8 to P28 in SOL muscle (Figure 3B). The FNDC5/irisin level was age-dependent in both groups (CTRL: *p* < 0.0001; SMR: *p* = 0.0120, Kruskal–Wallis). The FNDC5/irisin level was the maximum at P15 and then decreased to reach a minimum value at P28.

**Figure 4:**
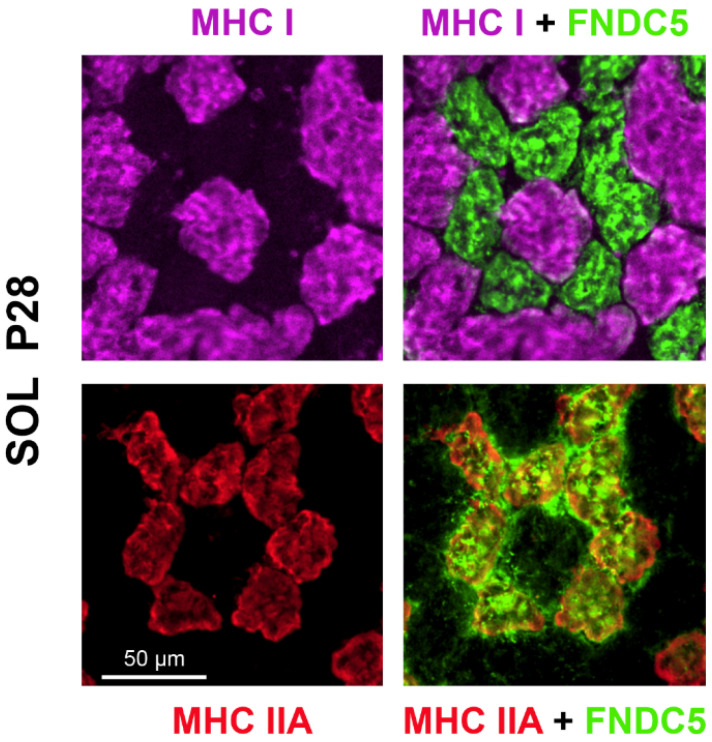
Differential distribution of FNCD5/irisin in SOL muscle at P28, as determined by immunohistochemistry. Muscle fibers were identified in SOL muscle by the expression of MHC isoforms, and sections were co-stained for FNDC5/irisin. Only type IIA fibers present a strong signal.

To determine which fiber type expressed FNCD5 in the SOL muscle, P28 muscle sections were immunolabeled for the different myosin heavy chains (MHC) and co-stained for FNCD5. Figure 4 shows a differential distribution of FNDC5/irisin between myofibers with expression exclusively in IIA fibers.

### 2.3. SMR Increased Irisin Levels in Plasma and CSF

**Figure 5:**
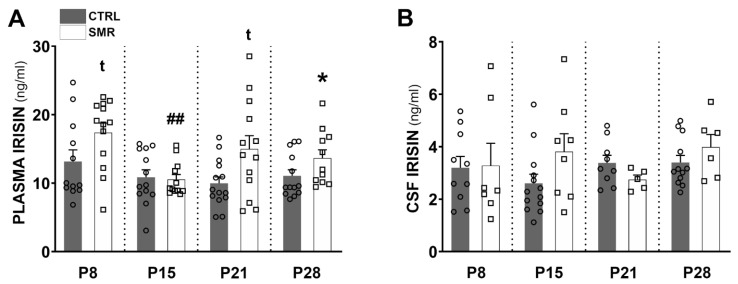
Effects of SMR on plasma and CSF irisin concentrations. (**A**) Plasma and (**B**) CSF irisin concentrations were determined by ELISA (ng/mL) in samples taken at P8, P15, P21 and P28. Each point represents the value for a given animal. Results expressed as mean ± S.E.M. Values were compared by a Mann–Whitney test. * *p* < 0.05 vs. CTRL; t: trend vs. CTRL; ## *p* < 0.01 vs. P8.

Irisin levels in plasma and CSF were compared with ELISA assays. In plasma, irisin levels vary between 3 and 30 ng/mL. The level remained stable with age in CTRL animals (Kruskal–Wallis *p* = 0.3997). On the other hand, in SMR animals, variations were observed (*p* = 0.0097), with a significant difference between P8 and P15 (Dunn’s multiple comparison test: *p* = 0.0023). A comparison of the CTRL and SMR values revealed an increase in the irisin level at P8 (+32%, *p* = 0.0678), P21 (+50%, *p* < 0.05 *p* = 0.0609) and P28 (+24% *p* = 0.0257), compared to age-matched controls. At P15, no difference (−3%, *p* = 0.8517) was observed between the CRTL and SMR animals (Figure 5A). In CSF, the irisin levels were between 1 and 8 ng/mL. No significant differences nor trends were observed for SMR with respect to age-matched CTRL (Figure 5B), nor between ages within one group.

### 2.4. SMR Had a Differential Impact on FNDC5/Irisin and BDNF Levels Depending on the Cerebral Structures

**Figure 6:**
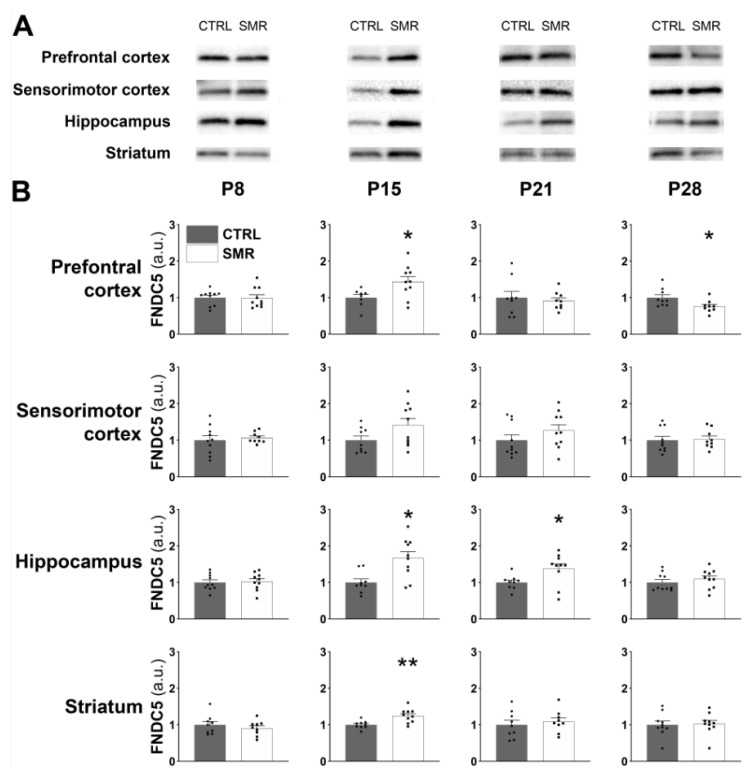
Effects of SMR on FNDC5/irisin levels in brain structures at different ages. Brain FNDC5/irisin level was determined by Western blotting (**A**). FNDC5/irisin expression was calculated as the ratio FNDC5/whole proteome (stain free) (**B**). a.u. = arbitrary unit. Each point represents the value for a given animal. Results expressed as mean ± S.E.M. *, *p* < 0.05; **, *p* < 0.01 vs. CTRL (Mann–Whitney).

**Figure 7:**
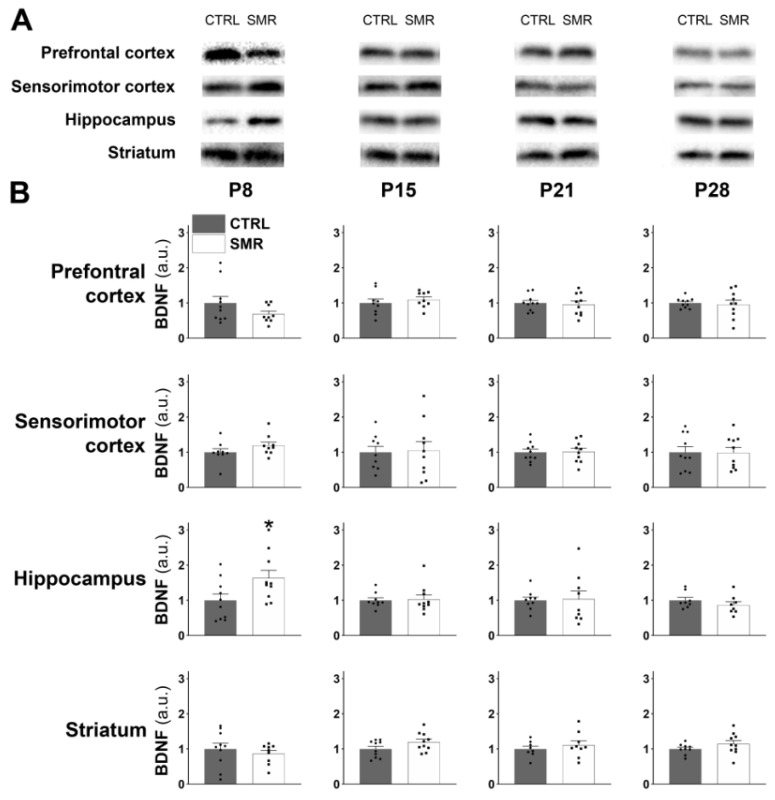
Effects of SMR on BDNF levels in brain structures at different ages. Brain BDNF level was determined by Western blotting (**A**). BDNF expression was calculated as the ratio BDNF/whole proteome (stain free) (**B**). a.u. = arbitrary unit. Each point represents the value for a given animal. Results are expressed as mean ± S.E.M. *, *p* < 0.05 vs. CTRL (Mann–Whitney).

FNDC5/irisin expression (Figure 6A) as well as BDNF (Figure 7A) expression were detected by Western immunoblotting in the prefrontal cortex, sensorimotor cortex, hippocampus and striatum at P8, P15, P21 and P28. Regarding FNDC5/irisin levels at P8, no significant difference was observed between CTRL and SMR for the four brain structures. At P15, a significant increase in FNDC5/irisin was observed in the prefrontal cortex (*p* < 0.05), hippocampus (*p* < 0.01) and striatum (*p* < 0.01) of SMR animals, but surprisingly, no significant difference was found in the sensorimotor cortex. At P21, the FNDC5/irisin level was significantly increased in the hippocampus of SMR animals (*p* < 0.05), while no change was observed in the other structures. Finally, at P28, FNDC5/irisin expression was significantly reduced in the prefrontal cortex of SMR animals (*p* < 0.05), with no significant difference between the two groups for the other cerebral structures (Figure 6B).

The comparison of males and females reveals a difference in the sensorimotor cortex at P15 and P21, with a higher expression level in females (*p* < 0.05 and *p* < 0.01, respectively). In contrast, values were lower in females in the striatum at P21 (*p* < 0.01) (see Supplementary Table S3).

Regarding BDNF levels, no differences were observed between CTRL and SMR, except in the hippocampus where BDNF expression was significantly increased for SMR animals only at P8 (*p* < 0.05) (Figure 7B). In addition, no sex effect was detected (Supplementary Table S4).

## 3. Discussion

Our objective was to determine whether SMR in rat pups alters the FNDC5/irisin expression in various hind limb muscles, brain structures and fluids throughout development from P8 to P28. The current investigation demonstrated that early SMR increased FNDC5/irisin levels specifically in the SOL muscle, from P21 onwards. Within the brain, SMR affected FNDC5/irisin expression in several structures as early as P15. The reported changes were always toward an increase in the FNDC5/irisin level, except in the prefrontal cortex at P28 where a decrease was observed. Since FNDC5/irisin mediates the BDNF expression levels, we also determined BDNF levels within the brain, and reported an increase only in the hippocampus at P8.

### 3.1. Methodological Considerations

The duration of immobilization was limited to 16 h per day and the cast was removed for 8 h during the light period to facilitate access to nipples and food intake, to allow pups to receive maternal care, and to avoid any feet injury. Thus, the immobilization was not continuous, potentially diminishing its impact. In addition, maternal behavior, such as anogenital licking or attempt to remove the cast, may induce movement of the pups’ hindlimbs and, in consequence, may also influence the effectiveness of immobilization. Consequently, our model should not be seen as replicating strict immobilization such as casting in children, for example, but is more relevant to describing the effects of neurodevelopmental disorders associated with low levels of physical activity and poor somatosensory interaction with the environment.

### 3.2. FNDC5/Irisin Levels Are Increased in SMR Pups

Myokines are secreted by skeletal muscles in response to physical activity [12] and are therefore also called “exerkines”. Previous studies provided evidence for an increase in the expression of this molecule in response to muscle contraction. Circulating levels were increased after a 3-week training course in a running wheel [12] or a 8-week swimming training course in adult rodents [21]. Exercise also upregulated Fndc5 mRNA in the quadricep muscles of adult mice that had access to a running wheel [22], whereas mRNA levels were decreased in response to hindlimb unloading [23]. Surprisingly, our study did not report a decrease in the FNDC5/irisin protein level in SMR rats, and the increased expression in SOL muscle, plasma and various cerebral structures in response to SMR did not fit our working hypothesis.

This FNDC5/irisin increase in the SOL appears from P15, the age at which animals almost present a mature posture. The immobilization of the hindquarter in an extended position in SMR animals prevents postural support and induces severe muscle atrophy. In view of muscular atrophy, we can suspect that postural control during the 8 h period of resumed activity requires a higher muscular demand. In addition, it has been shown previously that SMR pups develop toe walking due to ankle overextension. Their postural and locomotor features suggest the presence of spasticity [8]. Thus, the unexpected increase in the FNDC5/irisin level in SMR rats might be the consequence of the paradoxical increased activity of SOL during the 8 h period of activity.

Beyond muscle activity per se, Tsuchiya et al. [24] have shown that the increase in the irisin level was greater during exercise associated with marked muscle damage than during exercise with less muscle damage. It cannot be excluded that hindlimb movements caused fiber injury in the SOL immobilized in the shortened position. To sustain this hypothesis, Delcour et al. [8] have reported increased Pax7 labeling in the SOL agonist gastrocnemius, suggesting an increase in satellite cells in response to SMR.

Noteworthily, increased plasma levels have already been reported in humans after a 14-day episode of strict inactivity (bed-rest) [25]. These authors supported the idea that FNDC5/irisin is synthetized by adipose tissue rather than muscle. Similarly, in rats, a recent study showed a higher level in sedentary animals than in exercised ones, but the study was conducted in obese rats [26], which displayed higher levels of FNDC5/irisin than control rats, suggesting that FNDC5/irisin also originated from adipose tissue. Yet, an increased production from adipose tissue cannot explain why FNDC5/irisin levels were increased specifically in SOL muscle and not in EDL or TA.

Finally, it is unlikely that the increased FNDC5/irisin level is due to the higher activity of the pups during the 8 h period of activity since the myokine levels should thus be increased in all muscles, but not specifically in the slow-twitch postural SOL, especially since EDL and TA muscles have more IIA fibers than SOL.

### 3.3. Kinetics of FNDC5/Irisin Expression Level

We have shown that the FNDC5/irisin level in the SOL varies greatly during development, with low levels at birth and at P28 and a peak at P15. Such a bell-shaped curve is also observed in the rat L6 myoblast cell line grown in culture [27] or in cardiac muscle in vivo, where PGC1α, a factor involved in myokine secretion, is expressed [28]. An interesting finding is the peak expression around P15. The second postnatal week is considered a critical period for motor development, during which young rats are most sensitive to disuse [29]. This corresponds to the elimination of polyinnervation [30,31] and to the acquisition of a mature posture and complex motor acts [32]. We [33] and others [34] have shown that motor maturation is delayed in animals subjected to hindlimb movement restriction. For instance, postural control is achieved at P15 in control rats, but delayed until P20 in SMR animals [33]. We cannot exclude the hypothesis that the kinetics of FNDC5/irisin secretion are also delayed, with a peak occurring a few days later in SMR than in CTRL rats.

Indeed, in SMR pups, we observed an initial increase of irisin in plasma at P8, independent of SOL FNDC5/irisin expression, and a second increase occurred at P21/P28, correlating with the increased FNDC5/irisin level in SOL muscle. Since IIA myofibers exhibit the highest FNDC5/irisin expression level at P28, it could conceivably be hypothesized that myokine expression follows phenotypic maturation. In fact, it is yet not clear whether muscle maturation determines the irisin secretion level or depends on the FNDC5/irisin level. This specific localization is in accordance with the highest content of peroxisome proliferator-activated receptor gamma coactivator-1 alpha (PGC-1α), which is known to trigger Fndc5 expression in type IIA fibers [35,36]. Several studies indicate that SOL myofibers exhibiting an MHC IIA isoform are almost absent at birth (0–2% of muscle fibers) [37,38,39] and that their number gradually increases to reach a plateau at around P20. The kinetics of FNDC5/irisin expression do not follow the curve of IIA fibers’ appearance. Consequently, the maturation of the muscle phenotype does not appear to be the only factor that explains the increase in the FNDC5/irisin level. On the other hand, a recent study performed on C2C12 cells suggests that FNDC5/irisin might be involved in the regulation of the muscle fiber type [40]. The overexpression of FNDC5/irisin in C2C12 myotubes increased MHC IIA mRNA, whereas a downregulation decreased its expression. We have shown that SOL muscle from SMR rats has a higher proportion of MHC IIA at P28 [7]. This could be explained by the increased expression of FNDC5/irisin in this muscle during the first weeks after birth, considered as a critical period for attaining the adult MHC phenotype [37].

### 3.4. Muscle–Brain Dialogue

Besides its direct role on muscle, irisin is also well known to exert its effects on the brain and is now considered as one of the molecules mediating the beneficial effects of exercise on the brain [16,17]. The increase in the FNDC5/irisin level at P15 in SMR pups in several brain areas is likely to be of a peripheral origin since circulating levels are also enhanced. We did not detect any change in irisin levels in the CSF. However, these data must be interpreted with caution because very low levels were detected in both groups (approximately 3 ng/mL), i.e., close to the detection limit of the ELISA kit. However, endogen production by the brain itself cannot be excluded. Indeed, a 30-day endurance training course in mice resulted in the increased expression of Fndc5 and Bdnf genes in the hippocampus [20], suggesting that FNCD5 is also expressed in the brain.

One striking result is the lack of change in FNDC5/irisin level within the sensorimotor cortex. Noteworthily, daily casting pups’ hindlimbs severely reduced in their limb movements and somatosensory input (both tactile and proprioceptive). Consequently, the topographical organization of both sensory and motor cortices and neuronal properties were degraded, and the glutamatergic neurotransmission was increased as well [9].

The structure that displayed the greatest changes was the hippocampus, a structure involved in learning and memory. The hippocampus is particularly sensitive to the beneficial effects of exercise and several studies have provided evidence for morphological changes, such as the facilitation of long-term synaptic potentiation (LTP)-related pathways and increased neurogenesis, and suggested that FNDC5/irisin mediates these benefits [41].

We also performed BDNF assays in the brain, as irisin is known to induce BDNF expression [20]. We observed a small increase in the hippocampus at P8, which could be caused by the increased plasma level. However, at later ages (P21 and P28), there was no change in the cerebral BDNF despite the increase in the irisin plasma level. The stability of the BDNF level in brain areas may be explained by the fact that BDNF expression is regulated by other endocrine factors (such as insulin-like growth factor 1—IGF-1) and also by neuronal activity and cerebral blood flow [42]. For instance, in adult rats, a decrease in sensorimotor activity is accompanied by a decrease in the brain IGF-1 level [43], another factor known to induce BDNF expression [44]. Moreover, Park et al. [45] have shown that increased blood lactate released from skeletal muscle may induce hippocampal BDNF expression. It is, therefore, conceivable that the muscle–brain dialogue may not be fully effective during early development. Taken together, further work is needed to fully understand the muscle–brain dialogue during disuse and especially during development.

To conclude, our results suggest that SMR at the early stage of development affects the FNDC5/irisin level in a muscle involved in postural activity, but has only limited effects on BDNF expression in the brain. The increase in FNDC5/irisin expression in the SOL could be considered as a homeostatic mechanism to limit the deleterious consequences of early SMR. Indeed, it is well established that in adult mice, FNDC5/irisin exerts a trophic effect on the muscle in an autocrine manner, increasing muscle mass and strength [46], although Farrash et al. [47] reported no anabolic effect in response to FNDC5/irisin overexpression by an electrotransfer. Our results support the involvement of myokines in muscle function, but whether myokines play a role in cerebral BDNF modulation and in cognitive function during development should be investigated. Then, further studies are necessary to evaluate the impacts of RSM on motor activity in cognitive function.

## 4. Materials and Methods

### 4.1. Animals

Experiments were performed on CD-Sprague–Dawley rats purchased from Charles River Laboratories (L’Arbresle, France). Animals were housed in standard conditions (51% humidity, 22 °C and 12 h light–dark cycle), with access to food and water ad libitum. Following a 7-day acclimatization, a male was placed in a cage with 2 females each evening. Litters were obtained as already described [33]. Briefly, after mating with a male rat, pregnant female rats were housed in individual cages. At parturition (P1), litter size was adjusted to 10 pups per dam and litters were assigned to control (CTRL) or sensorimotor restriction (SMR) group. Thus, all pups within one litter were devoted to the same group. Body weight of pups was assessed daily from P1 to P28.

Given that maternal behavior can affect pup movements, we took care to include pups from at least two different litters on each day of analysis. We used a total of 144 CTRL and 138 SMR rats, obtained from, respectively, 18 and 18 litters. At P8, P15 and P21, muscle and brain data were obtained from 20 pups (10 CTRL from 2 litters and 10 SMR from 2 litters) for each developmental stage, for a total of 60 rats from 12 litters. At P28, muscle and brain samples were issued from different animals. Muscles were obtained from 16 CTRL rats and 15 SMR ones (3 and 3 litters, respectively), whereas brain samples were taken from 10 CTRL and 10 SMR rats (from 2 and 2 litters, respectively), for a total of 51 rats and 8 litters at P28. Blood and LCS samples were taken from some rats used for muscle analysis, but sometimes we had no sample or the volume was too small. We therefore used samples obtained from other rats of the same litters or from additional litters. The number of additional rats for CTRL group is 27 at P8 (4 litters), 23 at P15 (4), 9 at P21 (2) and 29 at P28 (4) and for SMR group, 26 at P8 (4 litters), 26 at P15 (5), 18 at P21 (3) and 13 at P28 (3).

### 4.2. HindLimb Immobilization

In the SMR group, pups were subjected to transient hindlimb immobilization from P1 to P28 for 16 h/day during the dark phase [33]. Briefly, hindlimbs gently bounded together with medical tape were immobilized in extended position from 16 PM to 8 AM with a cast, whose size was adapted to the pup growth. After casting, pups were returned to their mother. The casts did not prevent pups from urinating, defecating or receiving maternal care. The casts were then removed from 8 AM to 16 PM so that pups could move freely for 8 h/day. Protocols were optimized in order to minimize mother/pups separation, which did not exceed 15 min per day. CTRL pups were also separated from the mother and handled in equal proportions.

### 4.3. Tissue Sampling

Samples were taken at different ages: P8, P15, P21 and P28. After anesthesia with isoflurane (3% induction in 1.4 L/min air), animals received a lethal injection of T61 (MSD Animal Health) (0.3 mL/kg of body weight, intraperitoneal). CSF was collected using a syringe from the cerebellar–medullary cistern. Blood was collected in heparinized tubes directly by cardiac puncture after the opening of the thoracic cavity. Rats were exsanguinated with an intracardiac perfusion of 0.9% ice-cold NaCl. After craniotomy, sensorimotor cortex, prefrontal cortex, hippocampus and striatum, as well as soleus (SOL), extensor digitorum longus (EDL) and tibialis anterior (TA) muscles, were removed, weighed and placed in cryotubes and directly frozen in liquid nitrogen. CSF, muscle and brain samples were stored at −80 °C. After centrifugation of the blood samples (4000 rpm, 10 min, 4 °C), plasma was collected and stored at −20 °C.

### 4.4. Protein Isolation

Muscle samples were pounded into powder. Muscle and brain samples were weighed. Samples were introduced into a RIPA solubilization buffer (10 mM Tris/HCl, pH 7.4, 150 mM NaCl, 1 mM EDTA, 1% T-X100, 0.5% Na+ deoxycholate, 0.1% SDS) containing a cocktail of anti-proteases (EDTA-free protease inhibitor cocktail, cat# 11873580001, Roche, Basel, Switzerland) and anti-phosphatases (PhosStop, cat# 04906837001, Roche), according to 10 μL of buffer/mg of tissue. Sonication was then carried out on all the cell lysates before homogenization for 1 h at 4 °C and with slow stirring. After centrifugation (13,000 rpm, 10 min, 4 °C), the supernatants (protein fractions solubilized from the tissues) were collected and stored at −20 °C. Protein concentrations were calculated using the Bradford method (Protein Assay Dye Reagent Concentrate, cat#5000006, Biorad, Marnes-La-Coquette, France).

### 4.5. SDS-PAGE and Western Blotting

Solubilized protein fractions from tissues were diluted in Laemmli buffer (Tris/HCl 62.5 mM, pH 6.8, 10% glycerol, 2% SDS, 5% β-mercaptoethanol, 0.02% bromophenol blue) and denatured at 95 °C for 10 min.

Proteins were separated on pre-cast polyacrylamide gels (Criterion TGX Stain Free, Any kD, 18 well, cat#5678124, Biorad) with migration buffer (Tris base 25 mM, Glycine 190 mM, SDS 0.1%) at a constant voltage of 280 mV. Proteins were visualized after activation by an UV imager (ChemiDoc MP, Biorad). Image obtained was used to standardize results obtained following Western blots.

For muscle samples, protein transfer was carried out on a 0.2 μm nitrocellulose membrane (Trans-Blot^®^ TurboTM RTA Midi Nitrocellulose Transfer Kit, cat#1704271, Biorad) with a constant voltage of 25 V for 10 min in a Transblot Turbo Transfer System (cat# 1704150, Biorad). For brain samples, protein transfer was carried out on a 0.2 μm polyvinylidene fluoride membrane (Immun-Blot^®^ PVDF Membrane, cat#1620175, Biorad) in a transfer buffer (NaHCO_3_ 10 mM, Na_2_CO_3_ 3 mM, methanol 20%) by applying a constant amperage of 200 mA for 2 h with stirring and in a tank connected to a cooling system. For both techniques, transfer efficiency was then evaluated by acquiring the image of proteins with UV imager (ChemiDoc MP, Biorad).

After transfer, membrane was washed in TBS-Tween (Tris/HCl 15 mM, pH 7.6, NaCl 140 mM, Tween-20 0.05%) and then incubated in a saturation solution containing 5% non-fat dry milk in 0.05% TBS-Tween, for 1 h, at room temperature. Once saturated, membrane was incubated with the primary antibody, overnight, at 4 °C, under agitation: rabbit monoclonal anti-FNDC5 antibody (1:1000 diluted in 5% milk with TBS-Tween, ab174833, Abcam, Paris, France) or rabbit monoclonal anti-BDNF antibody (1:3000 diluted in 5% milk with TBS-Tween, ab108319, Abcam, Cambridge, UK). Antibody saturation and incubation conditions (dilution, time and temperature) were optimized for each antibody. Membrane was then washed 3 × 10 min with 0.05% TBS-Tween, under agitation. The secondary antibody was incubated for 2 h, at room temperature, under agitation: HRP anti-rabbit antibody (1:2000, diluted in 5% milk with TBS-Tween, 7074, Cell Signaling Technology, Ozyme, Saint-Quentin en Yvelines, France). Finally, membrane was washed again for 3 × 10 min with 0.05% TBS-Tween, under agitation.

Finally, membrane was incubated for 5 min, in the dark, in ECL reagent (Clarity^TM^ Western ECL Substrate, cat#1705061, BioRad). Acquisition of chemiluminescent signal was carried out with the Chemidoc MP imager. Signals obtained were analyzed and quantified using the Image Lab software 6.1 (Biorad). Normalization of protein signal intensities was carried out following quantification of respective total protein levels on Stain-Free images.

### 4.6. ELISA Assay

Blood and CSF irisin concentrations were analyzed using enzyme-linked immunosorbent assays (ELISA). Commercial ELISA kit was used (EK-067-29, Phoenix Pharmaceuticals, Strasbourg, France) following the manufacturer’s instructions. Since blood and CSF volume were sometimes not sufficient for analysis (in particular at P8), samples from 2 or 3 pups of the same litter were sometimes pooled.

### 4.7. Immunohistochemistry

Immunofluorescent staining was carried out on frozen muscle sections (10 μm). In summary, sections were incubated for 60 min at room temperature with BSA (Bovine Serum Albumine, Sigma Aldrich, Saint-Quentin-Fallavier, France) diluted to 3% in PBS (Phosphate Buffered Saline, Sigma Aldrich). They were then incubated for 60 min with a primary antibody cocktail containing FNDC5 (1:200, ab174833, Abcam) and MHC I or MHC IIA antibodies (1:200, BA-D5 or SC-71, respectively, DSHB) at 37 °C, followed by three rinses. Fluorescence-conjugated secondary antibodies (Alexa Fluor 488 and Alexa fluor 555 (1:250, A11008 and A21422, respectively, Thermofischer, Villebon-sur-Yvette, France) or Alexa fluor 647 (1:250, 1090-31, Southern Biotech, Clinisciences, Nanterre, France)) were applied for 30 min at 37 °C in obscurity, followed by three rinses. Finally, the sections were mounted in ProLong Gold Antifade Mountant (P36934, Invitrogen, Villebon-sur-Yvette, France) and coverslipped. Images were acquired using a Leica DMI8 inverted microscope, fitted with an automated motorized platform for mosaic imaging with the LAS X software (https://imillermicroscopes.com/pages/software-download accessed on 4 February 2024, Leica, Nanterre, France).

### 4.8. Data Analysis

Data normality was determined with the Shapiro–Wilk test. When normality and homogeneity of variance were satisfied, differences between experimental groups were analyzed by parametric *t*-test, ANOVA followed by Tukey post hoc test, or two-way ANOVA followed by Sidak multiple comparison test. Otherwise, a non-parametric Mann–Whitney or Kruskal–Wallis followed by Dunn’s post hoc test was used. n-values and statistical tests are reported in the figure legends. Outliers that were more than two standard deviations away from the mean were removed, as they likely resulted from technical errors. Results were expressed as the mean ± SEM. Statistical analyses were performed using Prism (version 7) software (Graphpad, San Diego, CA, USA). Values of *p* < 0.05 were considered statistically significant.

## Data Availability

The raw data supporting the conclusions of this article will be made available by the authors on request.

## Supplementary Materials

The following supporting information can be downloaded at: https://www.mdpi.com/article/10.3390/ijms25073918/s1.
