# Supplementary material for: Early Movement Restriction Affects FNDC5/Irisin and BDNF Levels in Rat Muscle and Brain
Source: Int J Mol Sci. 2024 Mar 31;25(7):3918. doi: 10.3390/ijms25073918 (PMC11011789; doi:10.3390/ijms25073918)
Supplement: Supplementary file 1 [file ijms-25-03918-s001.zip › SUPPL TABLE S1.pdf]

SUPPLEMENTARY TABLE S1. Overview of hindlimb muscle weight, by sex and group, and P-Value for sex and group effects.
Muscle Weight Wet/Body Weight ratio for SOL, EDL and Tibialis anterior muscles at P8, P15, P21 and P28.
Data are mean ± S.E.M. Values were compared with a two-way ANOVA with Tukey post-hoc test.
#p<0.05, ###p<0.001 vs. CTRL of the same sex.

		**CTRL male**	**CTRL female**	**SMR male**	**SMR female**	**P value (sex effect)**	**P value (group effect)**
SOL	P8	0.22 ± 0.01	0.19 ± 0.01	0.13 ± 0.01###	0.17 ± 0.01	0,7095	**0,0001**
	P15	0.31 ± 0.02	0.31 ± 0.03	0.21 ± 0.02#	0.21 ± 0.02#	0,8772	**<0,0001**
	P21	0.40 ± 0.02	0.43 ± 0.02	0.25 ± 0.02###	0.27 ± 0.01###	0,1716	**<0,0001**
	P28	0.43 ± 0.02	0.46 ± 0.03	0.26 ± 0.01###	0.28 ± 0.02###	0,2336	**<0,0001**
EDL	P8	0.27 ± 0.01	0.26 ± 0.01	0.27 ± 0.01	0.26 ± 0.01	0,2642	0,9807
	P15	0.33 ± 0.01	0.33 ± 0.02	0.35 ± 0.02	0.33 ± 0.02	0,6885	0,7463
	P21	0.45 ± 0.02	0.45 ± 0.01	0.33 ± 0.02###	0.37 ± 0.01###	0,1081	**<0,0001**
	P28	0.49 ± 0.01	0.50 ± 0.01	0.47 ± 0.02	0.45 ± 0.01	0,7755	**0,0221**
TA	P8	0.90 ± 0.02	0.88 ± 0.02	0.94 ± 0.03	0.97 ± 0.04	0,8953	**0,0207**
	P15	1.21 ± 0.03	1.19 ± 0.04	1.19 ± 0.03	1.12 ± 0.05	0,6746	**0,0427**
	P21	1.50 ± 0.04	1.53 ± 0.02	1.31 ± 0.04	1.36 ± 0.12	0,4954	**<0,0001**
	P28	1.82 ± 0.03	1.89 ± 0.04	1.62 ± 0.07#	1.61 ± 0.03###	0,7427	**<0,0001**
