# Supplementary material for: Early Movement Restriction Affects FNDC5/Irisin and BDNF Levels in Rat Muscle and Brain
Source: Int J Mol Sci. 2024 Mar 31;25(7):3918. doi: 10.3390/ijms25073918 (PMC11011789; doi:10.3390/ijms25073918)
Supplement: Supplementary file 1 [file ijms-25-03918-s001.zip › SUPPL TABLE S2.pdf]

SUPPLEMENTARY TABLE S2. Overview of FNDC5/irisine level in muscle, by sex and group, and P-Value for sex and group effects. FNDC5/Irisin level determined by western blotting and expressed relative to CTRL-Male. Data are mean ± S.E.M. Values were compared with a two-way ANOVA with Tukey post-hoc test. * : difference vs males of the same group.

**FNDC5/irisin**		**CTRL male**	**CTRL female**	**SMR male**	**SMR female**	**P value (sex effect)**	**P value (group effect)**
SOL	P8	1.00 ± 0.27	2.04 ± 0.49	2.34 ± 0.89	0.90 ± 0.35	0.7541	0.8763
	P15	1.00 ± 0.50	0.50 ± 0.27	4.79 ± 2.25	2.30 ± 1.10	0.3057	0.0696
	P21	1.00 ± 0.16	1.08 ± 0.29	2.80 ± 0.67	2.34 ± 0.42	0.6910	**0.0067**
	P28	1.00 ± 0.41	0.72 ± 0.12	1.69 ± 0.50	2.38 ± 0.54	0.6392	**0.0135**
EDL	P8	1.00 ± 0.15	0.68 ± 0.06	1.70 ± 0.28	0.96 ± 0.18	**0.0135**	**0.0213**
	P15	1.00 ± 0.07	0.90 ± 0.11	0.84 ± 0.14	1.21 ± 0.15	0.2956	0.5526
	P21	1.00 ± 0.08	0.59 ± 0.13	0.93 ± 0.20	0.35 ± 0.07 *	**0.0028**	0.2700
	P28	1.00 ± 0.07	1.11 ± 0.32	1.12 ± 0.36	1.26 ± 0.24	0.6701	0.6456
TA	P8	1.00 ± 0.40	0.90 ± 0.23	0.95 ± 0.41	0.92 ± 0.20	0.8406	0.9570
	P15	1.00 ± 0.09	1.53 ± 0.22	1.21 ± 0.18	1.61 ± 0.17	**0.0192**	0.4189
	P21	1.00 ± 0.13	2.25 ± 0.44	1.14 ± 0.42	1.32 ± 0.49	0.0968	0.3391
	P28	1.00 ± 0.27	1.46 ± 0.33	0.99 ± 0.23	1.33 ± 0.24	0.1597	0.7978
