# Supplementary material for: Early Movement Restriction Affects FNDC5/Irisin and BDNF Levels in Rat Muscle and Brain
Source: Int J Mol Sci. 2024 Mar 31;25(7):3918. doi: 10.3390/ijms25073918 (PMC11011789; doi:10.3390/ijms25073918)
Supplement: Supplementary file 1 [file ijms-25-03918-s001.zip › SUPPL TABLE S3.pdf]

SUPPLEMENTARY TABLE S3. Overview of FNDC5/irisine level in brain, by sex and group, and P-Value for sex and group effects. FNDC5/Irisin or BDNF level determined by western blotting and expressed relative to CTRL-Male. Data are mean ± S.E.M. Values were compared with a two-way ANOVA with Tukey post-hoc test. * : difference vs males of the same group; # : difference vs Ctrl of the same sex.

**FNCD5/irisin**		**CTRL male**	**CTRL female**	**SMR male**	**SMR female**	**P value (sex effect)**	**P value (group effect)**
Prefrontal cortex	P8	1.00 ± 0.07	0.97 ± 0.12	1.10 ± 0.13	0.86 ± 0.10	0.2217	0.9374
	P15	1.00 ± 0.18	1.24 ± 0.03	1.41 ± 0.23	1.82 ± 0.20	0.1084	**0.0218**
	P21	1.00 ± 0.34	1.00 ± 0.19	0.96 ± 0.15	0.88 ± 0.10	0.8503	0.6859
	P28	1.00 ± 0.08	1.10 ± 0.15	0.72 ± 0.07	0.87 ± 0.08	0.2725	**0.0308**
Sensorimotor cortex	P8	1.00 ± 0.22	0.91 ± 0.12	0.91 ± 0.03	1.10 ± 0.06	0.7064	0.7096
	P15	1.00 ± 0.19	1.29 ± 0.18	1.27 ± 0.22	2.00 ± 0.29	**0.0423**	**0.0481**
	P21	1.00 ± 0.05	1.81 ± 0.32	1.42 ± 0.11	2,13 ± 0.38	**0.0090**	0.1630
	P28	1.00 ± 0.14	0.81 ± 0.13	1.00 ± 0.10	0.87 ± 0.12	0.2253	0.8031
Hippocampus	P8	1.00 ± 0.12	0.95 ± 0.07	1.15 ± 0.06	0.84 ± 0.09	0.0663	0.7927
	P15	1.00 ± 0.11	0.85 ± 0.13	1.65 ± 0.26	1.40 ± 0.19	0.3110	**0.0065**
	P21	1.00 ± 0.10	0.89 ± 0.08	1.31 ± 0.22	1.28 ± 0.15	0.6579	**0.0409**
	P28	1.00 ± 0.09	1.09 ± 0.14	0.98 ± 0.12	1.31 ± 0.08	0.0769	0.3856
Striatum	P8	1.00 ± 0.12	0.81 ± 0.09	0.84 ± 0.05	0.82 ± 0.11	0.3119	0.4788
	P15	1.00 ± 0.05	0.92 ± 0.04	1.06 ± 0.05	1.32 ± 0.06 *,#	0.0982	**0.0006**
	P21	1.00 ± 0.11	0.58 ± 0.07 *	0.93 ± 0.10	0.72 ± 0.11	**0.0059**	0.7367
	P28	1.00 ± 0.12	0.88 ± 0.16	0.89 ± 0.15	1.04 ± 0.09	0.9360	0.8577
