# Supplementary material for: Early Movement Restriction Affects FNDC5/Irisin and BDNF Levels in Rat Muscle and Brain
Source: Int J Mol Sci. 2024 Mar 31;25(7):3918. doi: 10.3390/ijms25073918 (PMC11011789; doi:10.3390/ijms25073918)
Supplement: Supplementary file 1 [file ijms-25-03918-s001.zip › SUPPL TABLE S4.pdf]

SUPPLEMENTARY TABLE S4. Overview of BDNF level in brain, by sex and group, and P-Value for sex and group effects. FNDC5/Irisin or BDNF level determined by western blotting and expressed relative to CTRL-Male. Data are mean ± S.E.M. Values were compared with a two-way ANOVA with Tukey post-hoc test.

BDNF		CTRL male	CTRL female	SMR male	SMR female	P value (sex effect)	P value (group effect)
Prefrontal cortex	P8	1.00 ± 0.28	0.92 ± 0,25	0.57 ± 0.11	0.77 ± 0.19	0.7807	0.1934
	P15	1.00 ± 0.06	1.10 ± 0.28	1.12 ± 0.13	1.18 ± 0.09	0.6159	0.5289
	P21	1.00 ± 0.07	1.90 ± 0.14	1.13 ± 0.14	0.87 ± 0.15	0.5139	0.7267
	P28	1.00 ± 0.05	1.05 ± 0.09	1.00 ± 0.23	0.96 ± 0.13	0.9544	0.7526
Sensorimotor cortex	P8	1.00 ± 0.25	1.06 ± 0.05	1.29 ± 0.20	1.18 ± 0.10	0.8433	0.1978
	P15	1.00 ± 0.25	0.95 ± 0.23	0.89 ± 0.34	1.16 ± 0.39	0.7313	0.8720
	P21	1.00 ± 0.13	1.29 ± 0.13	1.11 ± 0.15	1.22 ± 0.18	0.1945	0.9131
	P28	1.00 ± 0.27	0.96 ± 0.20	1.09 ± 0.25	0.85 ± 0.16	0.5447	0.9516
Hippocampus	P8	1.00 ± 0.24	0.97 ± 0.29	1.65 ± 0.27	1.56 ± 0.36	0.8442	0.0512
	P15	1.00 ± 0.13	1.05 ± 0.07	1.13 ± 0.24	0.97 ± 0.09	0.7091	0.8748
	P21	1.00 ± 0.16	0.94 ± 0.08	0.91 ± 0.20	1.08 ± 0.40	0.8356	0.9248
	P28	1.00 ± 0.11	0.75 ± 0.04	0.77 ± 0.16	0.74 ± 0.07	0.1888	0.2854
Striatum	P8	1.00 ± 0.21	0.84 ± 0.23	0.76 ± 0.14	0.83 ± 0.11	0.7981	0.5200
	P15	1.00 ± 0.13	1.00 ± 0.10	1.26 ± 0.14	1.12 ± 0.10	0.5602	0.1179
	P21	1.00 ± 0.04	0.83 ± 0.11	1.14 ± 0.14	0.81 ± 0.12	0.0625	0.6429
	P28	1.00 ± 0.05	0.84 ± 0.06	1.12 ± 0.11	0.97 ± 0.14	0.1417	0.2451
